# Relationship between 24-h movement behaviors and frailty—a scoping review

**DOI:** 10.3389/fpubh.2026.1780746

**Published:** 2026-03-19

**Authors:** Yinhu Tan, Hang Li, Shuangxin Zhang, Xinchao Wang, Zhe Meng, Yixuan Liu, Yang Wang, Xiuling Zhou

**Affiliations:** 1 School of Nursing, Changchun University of Chinese Medicine, Changchun, Jilin, China; 2 School of Nursing, Guangxi University of Chinese Medicine, Nanning, Guangxi, China

**Keywords:** frailty, 24-h movement behaviors, moderate-to-vigorous physical activity, sedentary behavior, isotemporal substitution mode

## Abstract

**Background:**

Frailty is associated with increased risks of falls, disability, hospitalization, and mortality. The 24-h movement behaviors (24HMB) framework conceptualizes sleep, sedentary behavior (SB), light-intensity physical activity (LPA), and moderate-to-vigorous physical activity (MVPA) as mutually constrained components of daily time use and may inform frailty prevention and management.

**Objective:**

This scoping review maps evidence on associations between 24HMB and frailty and identifies methodological gaps to inform future research and nursing practice.

**Design:**

This review adheres to the Preferred Reporting Items for Systematic Reviews and Meta-Analyses extension for Scoping Reviews (PRISMA-ScR) and follows Joanna Briggs Institute (JBI) guidance.

**Methods:**

We searched PubMed, Embase, CINAHL, and Web of Science. We included observational studies of adults aged ≥18 years. Exposures were objectively measured or validated self-reported sleep, SB, LPA, and MVPA, including step counts, breaks in SB, isotemporal substitution models (ISM), and compositional data analysis (CoDA). Outcomes were frailty or prefrailty assessed using validated instruments. Quality was appraised with JBI tools.

**Results:**

Thirty-three studies showed good methodological quality. Longer SB, particularly prolonged, uninterrupted bouts, was associated with higher frailty. Greater MVPA was consistently associated with lower frailty. Light-intensity physical activity was generally beneficial but often attenuated when MVPA or total activity volume was modeled. Sleep fragmentation and poor sleep quality were associated with frailty. Isotemporal substitution models and compositional data analysis indicated that reallocating sedentary time to MVPA would yield the largest theoretical benefit, followed by reallocating to LPA. Higher daily step counts and more frequent or higher-intensity breaks in SB were associated with lower frailty.

**Conclusion:**

Evidence supports a 24-h integrated movement-behavior approach centered on MVPA, combined with reducing prolonged SB and improving sleep quality, for the prevention and nursing management of frailty.

**Systematic review registration:**

The study design and analytical protocol were prospectively registered on the Open Science Framework (OSF). The unique identifier is S39Y4, and the publicly accessible URL is https://doi.org/10.17605/OSF.IO/S39Y4.

## 1 Introduction

As a state of vulnerability in older adults caused by the decline of multiple physiological systems (1), frailty significantly increases the risks of falls (2), disability (3), hospitalization, and mortality (4). It is typically assessed using two major models. The first is the Fried Frailty Phenotype (Fried/CHS). This model identifies frailty based on five criteria—weight loss, weakness, slowness, low physical activity, and exhaustion—and demonstrates strong predictive validity (5). The second is the Frailty Index (FI), based on deficit accumulation. This model quantifies the proportion of health “deficits” (including symptoms, signs, functional limitations, and comorbidities) to represent an individual’s level of frailty (6). The prevalence of frailty is approximately 10.7% among community-dwelling older adults. It increases with age and is more pronounced in women (7). The prevalence among the global population aged 65 years and above reached 771 million in 2022 and is projected to account for 16% of the world’s total population by 2050, with the associated healthcare and caregiving burdens expected to increase significantly (8). Notably, the disease is not an irreversible, one-way degenerative process but rather a dynamic and potentially reversible condition (9).

Among the modifiable risk factors for frailty, physical activity and sedentary behavior (SB) show a consistent dose–response relationship with health outcomes. According to the 2020 WHO guidelines, older adults should engage in 150–300 min of moderate-intensity or 75–150 min of vigorous-intensity physical activity per week. They should also minimize and break SB as much as possible (10). A meta-analysis of multiple cohorts using accelerometer-based data further indicates that higher total activity volume and greater physical activity at all intensities are associated with a lower risk of all-cause mortality. In contrast, increased SB raises the risk of all-cause mortality. Notably, engaging in 30–40 min per day of moderate-to-vigorous physical activity (MVPA) can partially counteract the mortality risk associated with high SB (11).

The “24-h movement behaviors (24HMB)” framework proposed in recent years conceptualizes sleep — SB — light-intensity physical activity (LPA) — MVPA as a finite and interdependent set of time allocations. It also advocates for a balanced approach from a “whole-day” perspective. According to the Canadian 24-Hour Movement Guidelines for Adults and Older Adults, individuals are advised to obtain 7–8 h of high-quality sleep and to minimize and frequently break SB. They are also encouraged to engage in at least 150 min of moderate-intensity or 75 min of vigorous-intensity physical activity per week (12). This holistic perspective provides a new theoretical foundation for understanding the complex relationship between movement behaviors and frailty. It implies that interventions targeting frailty may need to be approached comprehensively by redistributing time across the 24-h day. Despite the growing number of studies adopting this framework and accumulating evidence indicating that prolonged SB and insufficient MVPA are significantly associated with frailty (13), the existing evidence remains fragmented and heterogeneous. Variations in devices, algorithms, wear sites, exposure structures, and tools used to assess frailty limit the feasibility of direct comparison and quantitative synthesis. Therefore, this study aims to systematically map the existing evidence on the relationship between 24-h movement behaviors and frailty through a scoping review, summarize consistent findings, and identify key methodological and evidential gaps. The findings will provide a foundation for future high-quality primary research (such as longitudinal cohort studies and intervention trials) and contribute to advancing relevant clinical and public health applications.

## 2 Methods

### 2.1 Design

This study is a scoping review conducted and reported in accordance with the Preferred Reporting Items for Systematic reviews and Meta-Analyses extension for Scoping Reviews (PRISMA-ScR) checklist. It is also guided by the latest methodological recommendations from the Joanna Briggs Institute (JBI) (14, 15). Its study design and analytical protocol have been prospectively registered on the Open Science Framework (OSF), accessible at: https://doi.org/10.17605/OSF.IO/S39Y4.

### 2.2 Define the research questions

① In the existing evidence on the relationship between 24-h movement behaviors (sleep, SB, LPA, and MVPA) and frailty in adults, how consistent are the research scopes, main findings, and direction of associations?
② What forms of heterogeneity exist in this field regarding measurement methods (e.g., devices, wearing protocols, behavioral definitions) and the tools used to assess frailty?
③ According to the isotemporal substitution model (ISM), how do theoretical differences in frailty risk arise when time is reallocated among various movement behaviors?
④ What specific and actionable implications can current evidence offer for frailty assessment and personalized activity interventions in nursing and clinical practice?

### 2.3 Research questions and framework (PCC: population, concept, context)

The research questions were formulated using the Population–Concept–Context (PCC) framework. Population: Adults aged 18 years and above were included, with no upper age limit, covering various life stages from middle to older adulthood. Studies focusing solely on older adults or specific subgroups (e.g., patients with hypertension, female cohorts) were also eligible and were stratified in the results. Concept: The 24-h movement behavior framework and its extended constructs, including sleep, SB, LPA, and MVPA, as well as related comparable metrics such as sedentary bouts/breaks, bouted and sporadic MVPA, total activity volume [e.g., Euclidean Norm Minus One (ENMO), total volume of physical activity (TVPA)], and step counts. Context: Community or general population settings, with no restrictions on countries, regions, or survey platforms.

### 2.4 Inclusion and exclusion criteria

#### 2.4.1 Study types

Included observational studies (cross-sectional, prospective, retrospective cohorts, and nested cohorts). Excluded randomized controlled trials of interventions, case series, case reports, methodological papers, and reviews.

#### 2.4.2 Participants

Adults aged 18 years and older (including community or general population cohorts. Disease-specific cohorts conducted in community settings were also included).

#### 2.4.3 Exposure

Any one or more items of sleep, SB, LPA, or MVPA measured using objectively wearable devices (hip/waist- or wrist-worn accelerometers, pedometers, etc.) or validated self-report instruments (e.g., International Physical Activity Questionnaires (IPAQ), time-use surveys, sleep scales). Studies reporting only step counts without corresponding SB/LPA/MVPA durations were classified under the “step-count exposure” subgroup.

#### 2.4.4 Outcome

Frailty, and—where explicitly reported and analyzed separately—pre-frailty, assessed using validated and published tools (such as the Fried/CHS and its adaptation/J-CHS, FRAIL/−J, Kihon Checklist, SOF, and the FI).

### 2.5 Literature search strategy

A systematic search of PubMed, Embase, CINAHL, and Web of Science was conducted from database inception through August 20, 2025. References and citation networks of the included studies were examined, and additional relevant studies not captured in the main search were identified through Google Scholar. Any refinements to the full search strategy and the inclusion/exclusion criteria were detailed in Appendix 1. The search and screening process was documented in accordance with PRISMA-ScR guidelines and illustrated in a flow diagram.

### 2.6 Study selection and data extraction

All search results were imported into EndNote X9. Duplicate records were first removed automatically using the “Find Duplicates” function, followed by manual verification for a second round of deduplication. Two reviewers (YHT and HL) then independently screened titles and abstracts, and after pilot calibration with a small sample, each record was labeled as “include/exclude/to be discussed.” Full texts of potentially eligible studies were then independently reviewed by both reviewers, with exclusion reasons pre-coded and documented (e.g., population not eligible, exposure not related to 24-h behaviors, failure to measure frailty, study design not relevant, insufficient information, or duplicate publication). Disagreements were initially resolved through discussion. If consensus could not be reached, a third reviewer (XLZ) made the final decision. The entire process—including identification, deduplication, title/abstract screening, full-text assessment, final number of included articles, and corresponding exclusion reasons—was reported following PRISMA-ScR guidelines and illustrated in a flow diagram. A standardized, pilot-tested extraction form was used to collect data, including author/year, country, study design, sample size (N), age group, device, wear site, 24-h movement behavior status (h/d), the relationship between 24-h movement behaviors and frailty, behavior components, frailty tool (preferably recorded in h/d; if the original text used min/d, values were converted uniformly), advanced metrics (such as sedentary breaks, prolonged sedentary bouts, bouted or sporadic MVPA, and total activity volume ENMO/TVPA), and the frailty assessment tool employed. The definitions of extracted fields and terminology adhered to the recommendations of JBI and PRISMA-ScR.

### 2.7 Quality assessment

Although scoping reviews do not aim to exclude studies based on quality assessment, a methodological appraisal was conducted to help interpret heterogeneity and assess the credibility of the evidence. The *JBI* tool was used to evaluate the quality of the literature: the *JBI* Analytical Cross-sectional (8 items) for cross-sectional studies and the JBI Cohort (11 items) for cohort studies. Two reviewers (SXZ and ZM) independently assessed each item as “yes/no/unclear/not applicable.” Prior calibration ensured consistency in standards, and any disagreements were resolved through adjudication by a third reviewer (YW).

## 3 Results

### 3.1 Results of literature screening and basic characteristics of included studies

**Figure 1:**
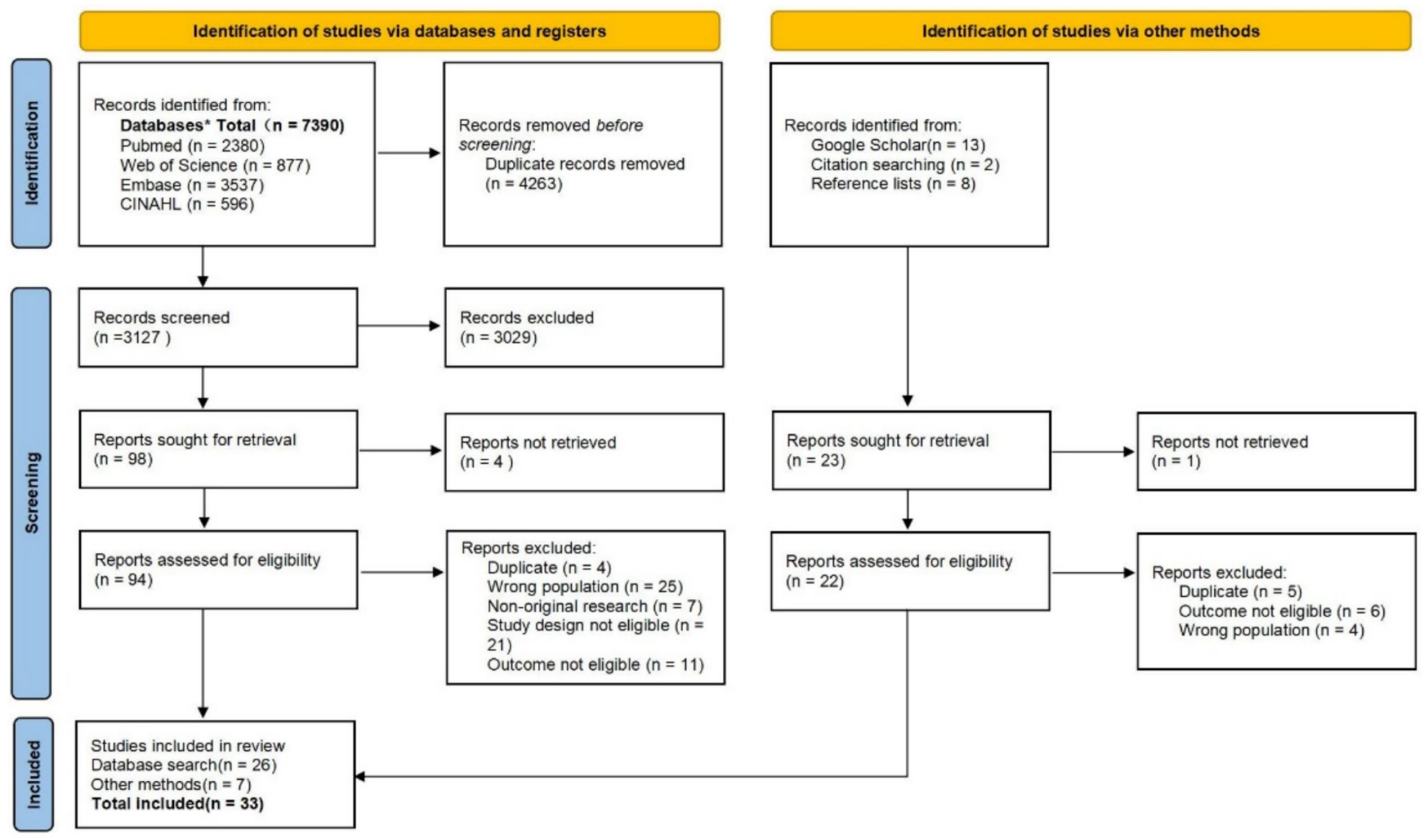
PRISMA flow diagram of the study selection process.

A total of 7,390 studies were identified through database searches, of which 3,127 remained after removing duplicates. Following title and abstract screening, 3,029 studies were excluded, and 26 were included after full-text re-screening. An additional 7 studies were identified through Google Scholar searches and relevant reference or citation tracing, resulting in a final total of 33 included studies. The screening flowchart is presented in Figure 1. Among the included studies, 9 originated from the United States (*n* = 9) (16–24), Japan (*n* = 10) (2, 13, 25–32), Spain (*n* = 6) (33–38), Brazil (*n* = 2) (39, 40), the United Kingdom (*n* = 1) (41), Korea (*n* = 1) (42), China (*n* = 3) (43–45), and Canada (*n* = 1) (46). The study designs comprised cross-sectional studies (*n* = 25) (13, 18, 19, 21–25, 27–31, 33–36, 39, 40, 42–47), and cohort studies (*n* = 7) (16, 17, 26, 32, 37, 38, 41), with one study employing a combined cross-sectional and cohort study approach (20). Sample sizes ranged from 186 to 78,508 participants. Additional study details are provided in Tables 1, 2.

**Table 1:** Characteristics of the Included Studies on 24-h activity behaviors and frailty.

Author	Country	Design	Sample size	Age groups	Relationship between 24 h activity behavior and decline in function	Behavior components	Frailty tool
Ensrud et al. (16)	United States	Prospective study	*n* = 2,505	Aged ≥67 years, average 75.7 ± 5.2 years.	Poor subjective sleep quality, WASO≥90 min, and nocturnal hypoxia (SaO₂ < 90% for ≥10% of sleep time) are associated with increased frailty risk, TST < 5 h, prolonged sleep latency, and low sleep efficiency have no significant association.	Sleep	FFP (5-item, CHS)
Blodgett et al. (46)	Canada	Cross-sectional study	*n* = 3,146	Aged ≥50 years, average 63.3 ± 10.1 years.	Individuals over 50 years old, especially those who are frail, commonly exhibit prolonged ST and insufficient MVPA, increases in SB and decreases in MVPA are independently associated with higher levels of frailty and adverse health outcomes.	SB, LPA, MVPA	Fried FI
Mañas et al. (35)	Spain	Cross-sectional study	*n* = 519	Aged ≥65 years, average 78.8 ± 4.6 years.	Replacing 30 min of SB with MVPA daily can significantly reduce frailty levels, substituting LPA for SB has an overall negligible effect, though it slightly benefits older adults with comorbidities. Both LPA and MVPA are associated with lower frailty levels in univariate or stratified models, while ST shows no significant relationship.	SB, LPA, MVPA	Frailty trait scale
del Pozo-Cruz et al. (33)	Spain	Cross-sectional study	*n* = 519	Aged 67–97 years, mean approximately 78.8 years.	Longer TST and a higher proportion of SB accumulated in segments of ≥10 min are associated with increased frailty, conversely, more frequent and longer interruptions in SB are related to lower frailty levels.	SB, LPA, MVPA	Frailty trait scale
Huisingh-Scheetz et al. (18)	United States	Cross-sectional study	*n* = 651	Aged 57–85 years, females average 71.3 years, males 72.5 years.	Each additional point in frailty score is associated with a 7% decrease in average hourly Counts Per Minute of activity, lower activity levels correlate with older age, increasing comorbidities, male gender, and higher BMI. Frail individuals exhibit delayed peak morning activity, with minor differences in evening activity.	Awake activity intensity measured in counts per minute (CPM).	Adapted fried phenotypic frailty scale (4 items, NSHAP dataset)
Nagai et al. (27)	Japan	Cross-sectional study	*n* = 886	Aged ≥65 years (mean age 73.6).	Replacing 30 min per day of SB with an equivalent duration of LPA reduces frailty risk by 14%, however, substitution with MVPA shows no significant effect after full adjustment and for those previously frail.	SB, LPA, MVPA	FFP (5-item, CHS)
Mañas et al. (36)	Spain	Cross-sectional study	*n* = 771	Aged ≥65 years, 76.8 ± 4.9 years.	Individuals who meet the MVPA guidelines (regardless of their sedentary levels) demonstrate better physical fitness and lower frailty levels. Conversely, among those who do not meet these standards, a higher LPA: SB ratio or less ST is also associated with lower frailty levels.	SB, LPA, MVPA	Frailty trait scale
Yuki et al. (32)	Japan	Prospective study	*n* = 401	Aged 65–82 years, average 71.1 ± 4.3 years.	Engaging in ≥5,000 steps per day or ≥7.5 min of MVPA significantly correlates with a lower risk of developing frailty.	Steps, LPA, MVPA	FFP (5-item, CHS)
Dantas da Silva et al. (39)	Brazil	Cross-sectional study	*n* = 457	Aged 60–96 years (mean age 70.3).	Using “≥150 min/wk. & < 540 min/d” as a reference, individuals with <150 min/wk. and ≥540 min/d exhibit a significantly increased prevalence of frailty.	PA level (≥150 min/week) combined with sedentary duration (≥540 min/day).	Adapted fried phenotypic frailty scale (4 items, NSHAP dataset)
Mañas et al. (37)	Spain	Prospective study	*n* = 186	Aged ≥65 years (baseline mean age 76.7 ± 3.9 years).	Among older adults who do not meet the PA guidelines, a higher frequency of SB interruptions is associated with a lower risk of frailty after four years, the lower the baseline frailty level, the more frequent future interruptions of SB.	SB, MVPA	FTS
Guida et al. (17)	United States	Prospective study	*n* = 615	Average age 71.5 years, stratified: 62–65 years 24.5%, 66–75 years 46.2%, ≥76 years 29.2%.	For every 10-point increase in the sleep fragmentation index, there is an increased risk of frailty after five years, however, TST shows no significant correlation with frailty risk.	Sleep	Modified fried FI
Mañas et al. (37)	Spain	Prospective study	*n* = 186	Aged 67–90 years, mean age 76.7 ± 3.9 years.	In physically inactive older adults, more frequent interruptions of SB at baseline are associated with a lower level of frailty after four years. Additionally, a lower baseline level of frailty predicts more frequent interruptions of SB in the future.	SB (including BST) / LPA / MVPA	Frailty trait scale
Kehler et al. (19)	United States	Cross-sectional study	*n* = 2,317	Aged ≥50 years, females: average age 67.88 years, males: 66.83 years.	In females, ST ≥ 30 min is associated with increased frailty, whereas higher average intensity of interruptions in SB is associated with lesser frailty, in males, sporadic MVPA helps alleviate frailty, while continuous MVPA is linked to a lower risk of frailty in all genders.	ST (Total, ≥30 min segments, Interruptions frequency/intensity/duration), MVPA (Segmented/Fragments)	Fried frailty index
Watanabe et al. (29)	Japan	Cross-sectional study	*n* = 3,616	Aged ≥65 years, average 72.3 ± 5.4 years.	An increase in daily SC significantly lowers the morbidity rate of frailty.	PAOnly (SC)	Fried frailty phenotype, Kihon checklist (KCL)
Higueras-Fresnillo et al. (34)	Spain	Cross-sectional study	*n* = 436	Aged 65–92 years (mean age 71.65 ± 5.28).	Increased MVPA, particularly “brisk walking,” is significantly correlated with a reduction in frailty scores, substituting 30 min/d of SB or LPA with “brisk walking” can decrease frailty scores by approximately 3.8–4.7 t-values.	Posture/Activity Subdivision: Lying, Semi-Reclining, Passive Sitting, Active Sitting, Standing, Slow Walking, Moderate Walking, Fast Walking, Other High Intensity, summarized as SB / LPA / MVPA.	FFP (5-item, CHS)
Chen et al. (47)	Japan	Cross-sectional study	*n* = 819	Aged 65–75 years, average age 70.9 ± 3.1.	The total amount of ST and its segmented/average duration are not significantly associated with frailty, however, total MVPA, segmented MVPA (≥10 min), and an increase in daily SC significantly reduce the risk of frailty. The optimal thresholds for differentiating between frail and non-frail individuals are 43.25 min/day (total MVPA), 9.13 min/day (segmented MVPA), and 3,841 steps/day (daily SC).	ST / LPA / MVPA (bouted & sporadic)/ Steps	FRAIL-J (Japanese version of the FRAIL scale)
Lefferts et al. (20)	United States	Cross-sectional study + prospective study	Cross-Sectio*n*al Study *n* = 427, Prospective Study *n* = 241	Aged ≥65 years, mean age approximately 72 ± 6 years.	Cross-sectional studies (*n* = 427, HTN) show that an increase in steps, particularly “aerobic steps,” is associated with a reduced risk of frailty, prospective cohort studies (*n* = 241, baseline non-frail, follow-up approximately 1.7 years) indicate that at least one daily instance of aerobic stepping (≥60 steps/min, continuous ≥10 min) significantly reduces the risk of new-onset frailty (HR ≈ 0.27), whereas total SC shows no significant correlation with the incidence risk.	SC / Step Rate, Cadence ≥60 steps/min defined as Aerobic Steps	Revised J-CHS
Kikuchi et al. (25)	Japan	Cross-sectional study	*n* = 511	Aged 65–84 years, mean age 73.4 ± 5.6 years.	Pre-frail/frail individuals have a lower proportion of MVPA and are more inclined towards prolonged SB (≥30 min), with less frequent short-duration SB, no significant differences in the proportion of LPA between groups.	SB (Short Bouts/Long Bouts) / LPA / MVPA	FFP (5-item, CHS)
Takamura et al. (28)	Japan	Cross-sectional study	*n* = 256	Aged ≥65 years.	Among those who do not participate in group sports, pre-frail individuals have significantly lower MVPA and SCs compared to non-frail individuals, among those who participate in group sports, there are no significant differences in MVPA and SCs between pre-frail and non-frail individuals, suggesting that community group sports may help pre-frail individuals maintain activity levels (significant interaction).	SB, LPA, MVPA	J-CHS
Wanigatunga et al. (23)	United States	Cross-sectional study	*n* = 638	Aged ≥70 years, average 77 years.	Increasing daily activities, reducing SB, and enhancing activity continuity can lower the risk of frailty, the fragmentation of SB has no significant impact on frailty risk.	Active minutes/day, Sedentary minutes/day, Total activity counts/day, Activity fragmentation, Sedentary fragmentation	FFP (5-item, CHS)
Li et al. (44)	China	Cross-sectional study	1,458	Aged ≥60 years (mean age 72.4).	More movement and less sitting can reduce the risk of frailty, ST ≥ 8 h/d poses the highest risk, and SB can diminish the protective effects of Physical Activity.	PA Total (MET-h/d, Light/Moderate/Vigorous), ST (h/d), Non-sleeping	FI
Martins et al. (40)	Brazil	Cross-sectional study	*n* = 456	Aged ≥60 years, mean age approximately 70 years.	Replacing 60 min of SB or sleep with Moderate PAdaily can reduce the morbidity rate of frailty by approximately 48%.	Sleep, SB, MPA, VPA (24 h Time Allocation, Self-reported)	SOF
Chang et al. (43)	China	Cross-sectional study	*n* = 214	Aged ≥65 years (mean age 80.8).	Individuals with frailty exhibit a significantly higher Number of sedentary segments per day. Total ST is notably longer under the mFFP and CFS-C criteria, while only the number of segments, not the total duration, is significant under SOF and CF-DC criteria.	SB (Total Duration, Bouts)	mFFP, CFS-C, SOF, CF-DC used concurrently.
Yokote et al. (30)	Japan	Cross-sectional study	*n* = 831	Aged 65–75 years.	Participation in MVPA, either independently or combined with exercise habits and social engagement, substantially reduces the risk of pre-frailty or frailty. The effects of exercise habits or social participation alone are not significant. Individuals engaging in MVPA exhibit reduced ST, with averages of 6.54 h per day versus 8.01 h per day for those less active.	MVPA (Accelerometer, ≥300 min/wk. MPA or ≥150 min/wk. VPA), Exercise Habits (≥30 min/day, ≥2 days/week, ≥1 year), Social Participation (≥1 time/week), STMeasured (Covariate)	FFP (5-item, CHS)
Li et al. (45)	China	Cross-sectional study	*n* = 1,099	Aged 60–70 years, females only.	Both sustained (≥10 min) and fragmented (<10 min) MVPA are significantly associated with a reduced risk of pre-frailty/frailty. Sustained LPA significantly lowers only the risk of pre-frailty.	SB (Total)/ LPA (Bouted, Sporadic)/ MVPA (Bouted, Sporadic)	FFP (5-item, CHS)
Tajima et al. (13)	Japan	Cross-sectional study	*n* = 1,004	Aged 85–89 years (median 86, IQR 85–88).	SB and limited MVPA (especially at higher intensities) are associated with a higher risk of (pre-)frailty. Replacing ST with 10-min intervals of MVPA is significantly associated with a lower risk of (pre-)frailty, however, replacement with LPA shows no significant association.	SB, LPA, MVPA, SC	Revised J-CHS
Yokote et al. (31)	Japan	Cross-sectional study	*n* = 811	Aged 65–75 years.	Insufficient MVPA significantly increases the risk of (pre-)frailty, independent of sleep quality. Among those meeting MVPA guidelines, poor sleep quality does not significantly relate to the incidence of (pre-)frailty.	MVPA (met/non-met), Sleep Quality (PSQI, good/poor), ST (h/d)	FFP (5-item, CHS)
Meister et al. (22)	United States	Cross-sectional study	*n* = 2,739	Aged ≥20 years, 20–64 versus ≥65.	Increased adherence to the 24-h guidelines is inversely related to the FI. Among individuals aged 20–64, the strongest association is with MVPA. For those aged ≥65, the strongest correlation is observed with “reduced screen time.”	MVPA, LPA, Sedentary Time, Recreational Screen Time, Sleep, Strength Training (6 components)	FI
Yang et al. (41)	United Kingdom	Prospective study	*n* = 78,508	Aged ≥45 years, average age 62.0 ± 7.8 years.	Within various FI levels, TVPA, MVPA, LPA are negatively associated with mortality risk, whereas ST is positively associated with risk. Life expectancy in frail individuals is most significantly impacted by low activity levels and high SB.	TVPA (TVPA, MVPA), LPA, ST	FI
Lee et al. (26)	Japan	Retrospective study	*n* = 387	Aged ≥65 years, mean age 72.0 ± 5.3 years.	In pre-frail elderly, higher TEE per day is associated with a greater likelihood of reverting to robustness within two years (a 24% increase in probability for every additional 100 kcal/day). No significant associations were found between SC, LPA, MVPA, or EEE and reversion to robustness.	SC, LPA (1.8–<3.0 METs), MVPA (>3.0 METs), EEE, TEE	Revised J-CHS
Liu et al. (21)	United States	Cross-sectional study	*n* = 5,726	Aged 18–64 years, average age 50.25 ± 0.31 years.	Meeting either two or three criteria of the 24-h guidelines significantly reduces the risk of frailty, with greater adherence correlating with lower risk. Meeting only one criterion does not show a significant effect.	MVPA, Sedentary, Sleep (all part of 24 h composition)	FI
Nam et al. (42)	Korea	Cross-sectional study	*n* = 2,650	Aged 70–84 years.	In community-dwelling older adults aged 70–84, substituting 10 min of ST with walking or MVPA significantly reduces the risk of frailty. Additionally, better neighborhood accessibility is associated with a lower risk of frailty.	SB, SC, MVPA	FFP (5-item, CHS)
Wingood et al. (24)	United States	Cross-sectional study	*n* = 726	Aged ≥65 years.	In individuals aged 65 and older, higher levels of activity fragmentation and lower capacity for short-duration high-intensity activities are associated with increased FI. Upon including all activity metrics, only activity fragmentation (positively correlated) and the intensity of the most active 10-min period (negatively correlated) are independently associated with frailty levels.	Total Activity (min/d), Non-active + Sleep (min/d), Number of Active Bouts, Bout Duration, Activity Fragmentation (1/Bout Duration), Maximum Intensity in 10 min (cpm), without distinction between LPA/MVPA, and without excluding sleep from non-active time.	FI

WASO: Wake After Sleep Onset TST: Total Sleep Time FI: Frailty Index MVPA: Moderate-to-Vigorous Physical Activity TEE: Total Energy Expenditure LPA: Light Physical Activity EEE: Exercise Energy Expenditure ST: Sedentary Time TVPA: Total Volume of Physical Activity SB: Sedentary Behavior PA: Physical Activity SOF: Study of Osteoporotic Fractures FTS: Frailty Trait Scale FFP: Fried Frailty Phenotype.

**Table 2:** Measurement tools, wear protocols, and activity/sleep outcomes of the included studies.

Author	Device	Wear site	24-h activity profile (h/d)
Ensrud et al. (16)	Devices: Wrist-based actigraph (Ambulatory Monitoring, Inc., AMI, ActionW2) and Home Polysomnography (PSG, Compumedics Safiro), Questionnaires: PSQI, ESS.	Arm: worn continuously for 5 nights/6 days (removed during bathing).	N/A
Blodgett et al. (46)	Device: ActiGraph AM7164 accelerometer.	Hip: worn during waking hours for 7 days, valid for ≥4 days, ≥10 h per day.	SB = 8.59 h/d, LPA = 5.40 h/d, MVPA≈ 0.26 h/d (15.3 min/d)
Mañas et al. (35)	Device: ActiGraph ActiTrainer.	Left hip: worn during waking hours for 7 days, valid for ≥4 days and each day ≥480 min.	SB ≈ 9.0 h/d, LPA ≈ 3.7 h/d, MVPA≈0.3 h/d
del Pozo-Cruz et al. (33)	Device: ActiGraph ActiTrainer.	Left hip: continuously for 7 days, valid day ≥480 min, ≥4 valid days.	SB ≈ 9.0 h/d, LPA ≈ 3.7 h/d, MVPA≈0.3 h/d
Huisingh-Scheetz et al. (18)	Device: ActiWatch Spectrum.	Non-dominant wrist: worn continuously for 72 h.	N/A
Nagai et al. (27)	Device: Actiband accelerometer (TDK), Questionnaire: Self-reported sleep.	Wrist: continuously worn for 14 days, sleep self-reported and excluded from SB.	Sleep = 6.7 h/d, SB = 8.50 h/d, LPA = 7.72 h/d, MVPA = 0.70 h/d
Mañas et al. (36)	Devices: ActiTrainer, ActiGraph wGT3X-BT.	Left hip, waking hours, 7 days, valid day ≥480 min, ≥4 valid days	ST ≈ 9.0 h/d, LPA ≈ 3.78 h/d, MVPA ≈ 0.32 h/d
Yuki et al. (32)	Device: Lifecorder uniaxial accelerometer (Suzuken).	Unreported wear location, continuously for 7 days, each day ≥10 h.	Steps = 7,204 steps/d, LPA ≈ 0.93 h/d, MVPA ≈ 0.34 h/d
Dantas da Silva et al. (39)	Questionnaire: IPAQ-LF.	N/A	PAL and ST: - PAL ≥ 0.36 h/d and ST < 9.0 h/d (31.4%) - PAL < 0.36 h/d and ST < 9.0 h/d (20.6%) - PAL ≥ 0.36 h/d and ST ≥ 9.0 h/d (24.2%) - PAL < 0.36 h/d and ST ≥ 9.0 h/d (23.7%)
Mañas et al. (37)	Device: ActiGraph accelerometer.	Hip-worn.	SB ≈ 8.84 h/d, MVPA ≈ 0.34 h/d, BST ≈ 71.7 times/d
Guida et al. (17)	Device: ActiWatch Spectrum, Questionnaire: Subjective sleep survey.	Wrist: continuously worn for 72 h.	TST ≈ 7.2 h/d, WASO ≈ 0.62 h/d, PS = 92.1%, SFI = 14.0
Mañas et al. (37)	Device: ActiGraph.	Left hip: worn during waking hours for a continuous week, valid day ≥8 h and ≥4 valid days.	ST ≈ 8.84 h/d, LPA ≈ 3.85 h/d, MVPA≈0.34 h/d, BST ≈ 71.7times/day, WT ≈ 13.02 h/d
Kehler et al. (19)	Device: ActiGraph 7,164.	Hip, awake hours, valid on ≥4 out of 7 days and ≥10 h per day	TST: - Females: 8.65 h/d, Males: 8.92 h/d - Sedentary bouts ≥30 min/day: Females: 3.38, Males: 3.86 - Sedentary interruptions/day: Females: 89.9, Males: 84.9 - Average intensity of interruptions (counts/min): Females: 391, Males: 442 - Average duration of interruptions (min): Females: 3.62, Males: 3.80 - Bouted MVPA (≥10 min): Females: 27.34 min/week, Males: 32.67 min/week - Sporadic MVPA (<10 min): Females: 38.62 min/week (≈5.52 min/day), Males: 69.66 min/week (≈9.95 min/day)
Watanabe et al. (29)	Device: Panasonic EW-NK52.	Waist: worn during waking hours, except during sleep/bathing/swimming.	≈ 4,081 steps/d
Higueras-Fresnillo et al. (34)	Device: Intelligent Device for Energy Expenditure and Activity (IDEEA) with multi-sensor pattern recognition.	Main unit around the waist, 5 sensors: anterior sternum, anterior aspects of both thighs, dorsal surfaces of both feet.	Awake time = 14.90 h/d, SB total = 7.13 h/d, LPA total = 7.32 h/d, MVPA total = 0.46 h/d
Chen et al. (47)	Device: Omron Active Style Pro HJA-350IT.	Waist: worn during waking hours for 7 days, valid day ≥4 and ≥10 h/day.	Total sedentary = 7.62 h/d, LPA = 5.68 h/d, MVPA total = 0.87 h/d
Lefferts et al. (20)	Device: Omron HJ-321.	Hip or pocket: removed during water activities/sleep.	Total steps = 5,445 steps/d, Aerobic steps = 1,457 steps/d
Kikuchi et al. (25)	Device: Omron Active Style Pro HJA-750C.	Hip: continuously for 7 days, valid day ≥10 h/day and ≥4 days.	Robust (*n* = 264): - WT: 15.09 h/d, SSB: 4.55 h/d, LSB: 2.79 h/d, Total sedentary time: 7.34 h/d, LPA: 6.77 h/d, MTHI: 0.98 h/d Pre-frail (*n* = 234): - WT: 14.48 h/d, SSB: 4.35 h/d, LSB: 3.10 h/d, Total sedentary time: 7.45 h/d, LPA: 6.24 h/d, MTHI: 0.79 h/d Frail (*n* = 13): - WT: 13.91 h/d, SSB: 3.85 h/d, LSB: 4.83 h/d, Total sedentary time: 8.68 h/d, LPA: 4.98 h/d, MTHI: 0.25 h/d
Takamura et al. (28)	Device: Active Style Pro HJA-750C (triaxial accelerometer, Omron).	Waist/hip: worn during awake hours, valid ≥10 h/day, ≥4 days, epoch = 1 min.	Participation in group sports: - Non-frail: SB 7.67 h/d (460.1 min/d), LPA 6.50 h/d (389.9 min/d), MVPA 0.91 h/d (54.5 min/d), Steps: 5553 steps/day - Pre-frail: SB 7.08 h/d (424.7 min/d), LPA 6.50 h/d (389.8 min/d), MVPA 0.93 h/d (55.7 min/d), Steps: 5090 steps/day - Non-participants (non-frail): SB 7.39 h/d (443.4 min/d), LPA 6.26 h/d (375.6 min/d), MVPA 0.94 h/d (56.4 min/d), Steps: 5625 steps/day - Non-participants (pre-frail): SB 7.64 h/d (458.6 min/d), LPA 5.82 h/d (349.3 min/d), MVPA 0.46 h/d (27.4 min/d), Steps: 3203 steps/day
Wanigatunga et al. (23)	Device: ActiGraph GT9X.	Non-dominant wrist: worn continuously for 7 days, removable for >30 min of swimming/bathing.	Average active time ≈ 6.4 h/d, ST ≈ 11.6 h/d, activity fragmentation ≈ 25%, sedentary fragmentation ≈ 13%
Li et al. (44)	No device, Questionnaires: International PAQuestionnaire – Short Form (IPAQ-SF), Single-item sitting time (ST).	N/A	Sitting time ≈ 4.64 h/d, PA ≈ 4.91 MET-h/d
Martins et al. (40)	Questionnaires: IPAQ, Pittsburgh Sleep Quality Index – Brazilian Portuguese version (PSQI-BR).	NA (self-reported questionnaire)	Moderate PA ≈ 0.75 h/d, Vigorous PA ≈ 0.07 h/d, TST ≈ 7.24 h/d, SB ≈ 7.12 h/d
Chang et al. (43)	Device: ActiGraph wGT3X+.	Waist: worn for 7 days, daily valid ≥10 h, ≥5 valid days.	Sedentary time: 609.74 ± 79.29 min/d (≈10.16 h/d), NOSB: 5.51 ± 2.09 times/d.
Yokote et al. (30)	Device: Omron Active Style Pro HJA-350IT (triaxial accelerometer).	Unspecified (reported wear duration standard).	None 480.5 min/d (8.01 h/d), Exercise 492.9(8.22 h/d), Social 484.4(8.07 h/d), MVPA 392.3(6.54 h/d), Ex+Soc 482.2(8.04 h/d), Ex+MVPA 433.1(7.22 h/d), Soc + MVPA 425.4(7.09 h/d), All 408.0(6.80 h/d)
Li et al. (45)	Device: ActiGraph wGT3X-BT.	Left hip: worn for 7 days, valid day ≥4 and ≥10 h/day.	WT ≈ 14.79 h/d, SB ≈ 9.13 h/d, LPA (bouted) ≈ 3.03 h/d, LPA (sporadic) ≈ 2.09 h/d, MVPA (bouted) ≈ 0.20 h/d, MVPA (sporadic) ≈ 0.35 h/d
Tajima et al. (13)	Device: Omron Active Style Pro HJA-750C.	Waist: worn during waking period (except during bathing/water exposure), continuously for 7 days.	Sedentary (≤1.5 METs): Non-frail: Males 8.75 h/d, Females 8.35 h/d, Pre-frail: Males 8.92 h/d, Females 8.85 h/d, Frail: Males 9.25 h/d, Females 9.02 h/d LPA (1.6–2.9METs) (graph-adjusted mean estimates): -Non-frail: Males ≈4.50 h/d, Females ≈5.67 h/d, Pre-frail: Males ≈4.15 h/d, Females ≈5.30 h/d, Frail: Males ≈3.50 h/d, Females ≈4.90 h/d MTHI (≥3.0 METs) (graph estimates): Non-frail: Males ≈0.37 h/d, Females ≈0.33 h/d, Pre-frail: Males ≈0.30 h/d, Females ≈0.27 h/d, Frail: Males ≈0.20 h/d, Females ≈0.18 h/d. Steps (steps/day) Non-frail: Males ≈4,500, Females ≈4,000. Pre-frail: Males ≈4,000 (about 500 less), Females ≈3,500 (about 500 less). Frail: Males ≈3,500 (about 1,000 less), Females ≈3,000 (about 1,000 less)
Yokote et al. (31)	Device: Active Style Pro HJA-350IT (triaxial accelerometer, Omron).	N/A	MVPA met + good sleep quality (MVA + SLP+): 6.87 h/d (412 min/d) MVPA met + poor sleep quality (MVA + SLP−): 6.96 h/d (417.8 min/d) MVPA not met + good sleep quality (MVA − SLP+): 8.12 h/d (486.9 min/d) MVPA not met + poor sleep quality (MVA − SLP−): 7.95 h/d (477.1 min/d)
Meister et al. (22)	Device: ActiGraph AM7164 (worn on hip, 1-min epoch), Questionnaire: Self-reported items (sleep, recreational screen time, strength training).	Right hip: worn during waking hours for 7 days, valid for ≥4 days and ≥10 h/day.	No mean values provided h/d, as per guidelines and compliance reporting: ST ≤ 8 h/d (compliance 47.0%), sleep 7–9 h (20–64)/7–8 h (≥65) (compliance 59.0%), MVPA≥150 min/week (36.4%), LPA “higher” (highest quartile by age) (25.1%), leisure screen ≤3 h/d (76.9%), strength training ≥2 times/week (18.7%)
Yang et al. (41)	Device: Wrist-worn accelerometer (UK Biobank).	Wrist	MVPA ≈ 0.52 h/d, LPA = 5.27 h/d, ST = 7.98 h/d
Lee et al. (26)	Device: Lifecorder uniaxial accelerometer (Suzuken).	Waist belt: worn continuously for 7 days (not worn during sleep/showering).	Steps≈7,811 steps/day, LPA ≈ 61.4 ≈ 1.02 h/d, MVPA≈0.37 h/d, EEE ≈ 182.6 kcal/d, TEE ≈1666.5 kcal/d
Liu et al. (21)	No device, Questionnaires: GPAQ, NHANES sleep items.	N/A	Sedentary = 6.36 h/d, Sleep = 7.06 h/d, MVPA ≈ 2.80 h/d
Nam et al. (42)	No device, Questionnaires: IPAQ, IPAQ-E.	N/A	Reported by frailty status (mean ≈ h/d)—SB: Robust 5.03 / Pre-frail 5.45 / Frail 7.50, Walking: 1.23/1.00/0.53, MVPA: 0.97/0.93/0.27 (converted from Table 1 min/d to h/d)
Wingood et al. (24)	Device: ActiGraph Insight accelerometer (24-h wear).	N/A	Active time ≈ 5.63 h/d, non-active + sleep ≈ 18.37 h/d

WASO: Wake After Sleep Onset TST: Total Sleep Time MVPA: Moderate-to-Vigorous Physical Activity TEE: Total Energy Expenditure LPA: Light Physical Activity EEE: Exercise Energy Expenditure ST: Sedentary Time PAL: Physical Activity Level SB: Sedentary Behavior EEE: Exercise Energy Expenditure WT: Wear Time PS: Percent Sleep BST: Breaks Sedentary Times SSB: Short Sedentary Bouts LSB: Long Sedentary Bouts MTOI: Moderate To High Intensity PA: Physical activity NOSB: Number of Sedentary Bouts SFI: Sleep Fragmentation Index.

### 3.2 Literature quality

The 33 studies included in this review were generally of good quality, as assessed using the JBI tool. Studies that employed objective accelerometer-based exposure measurements, standardized frailty assessment tools (e.g., Fried/CHS, J-CHS, or FI), and multivariable modeling were rated “Yes” on most items, indicating high overall quality. Common limitations included lower measurement reliability or inadequate control of confounding in studies using self-reported exposure measurements, incomplete sample representativeness, insufficient handling of loss to follow-up or time-varying exposures in cohort studies, and challenges in distinguishing sleep from sedentary behavior in certain 24-h protocols. Overall, cross-sectional studies consistently performed well on items such as “clear inclusion criteria, objective exposure measurements, standardized outcome scales, and appropriate statistical analysis.” Cohort studies were generally rated “Yes” for “consistent exposure measurements, adequate follow-ups, valid outcome assessment, and appropriate statistical analysis.” Nonetheless, the aforementioned reporting and measurement limitations should be considered when interpreting the overall strength of the evidence. Further details are provided in Appendix 2.

### 3.3 Activity measurement and frailty assessment

Among the included studies, 25 measured activity using objectively wearable devices, 5 relied mainly on subjective questionnaire assessments, and 3 adopted a mixed approach combining both objective and subjective measurements. The most common protocol involved wearing wrist- or hip-mounted accelerometers for 7 consecutive days, with valid days defined as ≥4–5 days of at least 8–10 h of wear time per day (19, 32, 33, 35, 37, 46). Seventeen studies assessed frailty using the Fried Frailty Phenotype (FFP), including CHS/J-CHS and its adapted versions; 8 used the FI, and 5 employed the Frailty Trait Scale (FTS). Other tools, such as FRAIL and SOF, were used in a few studies (40, 43, 47).

### 3.4 24HMB

Approximately 24 studies assessed SB. Among accelerometer-based studies, mean daily SB typically ranged from 7.34 to 11.60 h/d (23, 25), whereas studies using self-reported questionnaires reported 4.6–6.4 h/d (21, 44). Higher total SB—especially when accumulated in prolonged bouts (≥10–30 min)—was consistently associated with a greater prevalence of frailty. In contrast, more frequent breaks in sedentary time (BST) (i.e., sit-to-stand transitions) were linked to lower frailty levels (13, 19, 25, 33, 34, 36, 37, 40, 42–44, 46). Eighteen studies reported LPA, with average daily durations ranging from 0.93 to 7.72 h/d (27, 32). Overall, higher LPA levels were associated with a reduced risk of frailty, and bouted LPA was linked to a lower likelihood of frailty-related outcomes. However, after adjusting for MVPA or total activity volume in the same model, the independent association of LPA often weakened or became nonsignificant for MVPA (27, 35, 37, 45, 47). Twenty-four studies measured MVPA, with daily levels generally ranging from 0.20 to 0.98 h (25, 45). Evidence consistently indicated that higher MVPA was associated with a lower risk of developing frailty across different study designs and populations (19, 28, 30, 32–35, 40). Sleep was assessed in most studies using measures such as Wake After Sleep Onset (WASO), sleep efficiency, oxygen desaturation index, or the Pittsburgh Sleep Quality Index (PSQI). Reported total sleep time (TST) typically ranged from 6.7 to 7.06 h/day (21, 27). Poorer or more fragmented sleep (higher WASO, lower sleep efficiency) or increased nocturnal hypoxia was associated with higher levels of (pre)frailty (16, 17, 21, 27).

### 3.5 Compositional and substitution models, step counts and patterns, and guideline compliance

Both isotemporal substitution models and compositional data analysis models indicated that theoretically replacing SB with an equivalent amount of MVPA yielded the greatest benefit (13, 27, 34, 36, 40, 42). Substituting SB with an equivalent amount of LPA also showed positive effects, though to a lesser extent (13, 27, 36, 45). Regarding step counts, a higher number of daily steps was consistently associated with lower frailty levels (29, 32). Operational thresholds were identified—achieving ≥5,000 steps per day or ≥7.5 min per day of MVPA was linked to a reduced risk of incident frailty, and aerobic steps (i.e., continuous steps at higher cadence) demonstrated a stronger association with lower frailty compared with total steps (20, 29, 32). In terms of behavioral patterns, shorter prolonged sedentary bouts were associated with lower frailty. Additionally, increasing the frequency or intensity of sit-to-stand transitions was linked to reduced frailty (19, 25, 33, 37). Findings on the “fragmentation/integration” of activity were less consistent. Higher activity fragmentation was independently associated with higher frailty levels, even when multiple indicators were included in the same model (24). In contrast, sedentary fragmentation indicated no significant overall association with frailty (23). Regarding guideline compliance, among individuals not meeting physical activity guidelines, a higher baseline frequency of sit-to-stand transitions was associated with lower frailty at follow-up. However, this association was not significant among those meeting guidelines or in the overall sample (37). Gender- and pattern-specific differences were also observed: ≥30-min uninterrupted sedentary bouts were linked to higher frailty levels in women. Higher average intensity of sit-to-stand transitions corresponded to lower frailty in all genders. Moreover, greater amounts of sporadic MVPA were associated with lower frailty in men (19). Within the framework of 24-h movement behavior guidelines, adherence to a greater number of components showed a gradient association with lower frailty. Meeting MVPA recommendations typically represented the key determinant of this association (21, 22, 36, 37, 40).

## 4 Discussion

This review demonstrates that MVPA is inversely associated with frailty, whereas greater SB—especially prolonged sedentary bouts—is linked to a higher risk of frailty (34). These findings align with the conclusions of the 2020 WHO Guidelines on physical activity and sedentary behavior, which highlight that reducing ST and increasing MVPA are effective strategies for frailty prevention (10). Based on isotemporal substitution models and compositional data analyses, we also found that theoretically replacing sedentary time with an equivalent amount of MVPA yields the greatest benefit. In contrast, substituting sedentary time with LPA has a smaller effect, which is more dependent on an individual’s health status (35). This suggests that higher-intensity physical activity has a more profound impact on frailty prevention. Notably, although MVPA demonstrates preventive effects against frailty, our review indicates that these effects do not exist in isolation but interact significantly with other behavioral components. Among individuals with insufficient MVPA, high-frequency BST can still substantially reduce frailty risk (37). This implies that fragmented LPA may have a compensatory protective effect. These findings strongly support viewing the 24-h day as a dynamic and interrelated whole rather than merely a sum of separate time segments. It is also important to note that these effects may vary across age groups.

Notably, accumulating evidence indicates that the relevance of 24-h movement behaviors to frailty is not confined to late life, underscoring the importance of prevention prior to old age. Using NHANES 2005–2006 and stratifying participants into adults aged 20–64 years and those aged ≥65 years, Meister et al. (22) reported an inverse dose–response relationship in both strata, such that meeting a greater number of 24-h movement guideline components was associated with lower frailty level; moreover, the guideline component most strongly associated with lower frailty differed by age, with MVPA being most prominent in adults aged 20–64 years, whereas recreational screen time was most prominent in adults aged ≥65 years. In addition, Liu et al. (21) analyzed U.S. adults aged 18–64 years (NHANES 2007–2018) and found that adhering to combinations of guideline recommendations—such as meeting both MVPA and sleep recommendations, meeting both MVPA and sedentary-behavior recommendations, or meeting both sedentary-behavior and sleep recommendations—and particularly meeting two or three recommendations, was associated with lower odds of frailty across age strata (18–39, 40–59, and 60–64 years), although some associations varied by age (e.g., the joint MVPA–sedentary-behavior association was not significant in those aged 60–64 years). Taken together, these findings support a life-course perspective, suggesting that optimizing sedentary behavior, MVPA, and sleep across adulthood—not only in later life—may be important for shifting frailty trajectories, and that age-tailored behavioral targets may be warranted.

The impact of SB depends not only on “how long one sits” but also on how ST is accumulated (i.e., prolonged sedentary bouts) and broken. Several studies have shown that prolonged sedentary bouts of ≥10 min are significantly associated with frailty, whereas higher frequency and intensity of sedentary breaks are linked to a lower risk of frailty. This association is particularly pronounced in older women, for whom longer ST corresponds to a higher likelihood of frailty (19, 33). However, the relationship between sedentary fragmentation and frailty is not entirely consistent. Some studies report no significant link between sedentary fragmentation and frailty, yet the effect of SB is closely related to the manner and intensity of its interruptions (23). These findings suggest that, in practical nursing interventions, increasing the intensity of sedentary breaks may be more effective in reducing frailty risk than merely reducing the number of sedentary “bouts” (24).

In addition, we have found that poor sleep quality (such as increased WASO and reduced sleep efficiency) is significantly associated with frailty (16, 17). However, among individuals who already meet the MVPA recommendations, the relationship between poor sleep quality and frailty is significantly weakened. This suggests that daytime activity, particularly MVPA, can partially offset the negative effects of poor sleep quality (31). Furthermore, sleep quality and daytime activity behaviors may have a bidirectional relationship: fragmented sleep leads to daytime fatigue and reduced activity, while low daytime activity further disrupts nighttime sleep, creating a self-reinforcing vicious cycle. This interactive effect between daytime activity and sleep quality further underscores the importance of an integrated 24-h behavioral management approach. These findings align with the Canadian 24-Hour Movement Guidelines, which emphasize the complementary relationship between physical activity and sleep (12).

In summary, MVPA should be considered the primary target of frailty intervention. Increasing higher-intensity physical activity can effectively slow the progression of frailty, especially among older adults at earlier or mild stages of frailty progression. For populations with prolonged ST, nursing interventions should prioritize reducing prolonged sedentary bouts and increasing the intensity of sedentary breaks, e.g., encouraging older adults to engage in short bouts of brisk walking or standing activities throughout the day. Step counts should be used as a behavioral prescription tool, with 5,000 steps per day serving as an initial target for health management. Sleep management should be integrated with MVPA and sedentary interventions to create a comprehensive 24-h behavioral management strategy.

Translating studies on 24-h movement behaviors into practical clinical and public health applications remains a major challenge. Most current recommendations are still theoretical and lack validation of their effectiveness and feasibility in real-world settings, such as communities or outpatient clinics. Integrating complex behavioral assessments into routine health screening, while ensuring equitable access across different socioeconomic groups, remains an urgent issue. Future translational studies should focus on three key directions. First, developing clinical decision support systems that integrate multidimensional behavioral data (e.g., sedentary patterns and sleep quality) and link them with electronic health records, thereby providing a foundation for personalized health guidance. Second, designing and testing low-burden “behavioral micro-interventions” targeting behavior (such as activity snacks or standing breaks), leveraging mobile health technologies to improve resident adherence. Finally, promoting multisectoral collaboration and integrating health into all policies at the policy and environmental levels—through urban renewal, insurance payment model reform, and the development of social support networks—to create an enabling environment that supports active and healthy living.

## 5 Limitations

This review synthesizes findings from multiple studies. However, there are some inconsistencies in results due to methodological variations. First, differences in measurement standards, such as accelerometer wear sites and the cut-points used to define sedentary and active time, may have affected the accuracy of results. For instance, notable discrepancies exist between accelerometer wear sites (hip versus wrist) and in the definitions of sedentary bouts (e.g., ≥10 min vs. ≥30 min). These differences, in turn, influence the assessment of frailty risk. Second, variations in statistical modeling approaches (e.g., traditional regression versus isotemporal substitution models) may lead to differences in the interpretation of the association between MVPA and frailty. In isotemporal substitution models, the effects of SB and the benefits of MVPA are more clearly delineated, whereas traditional regression models may underestimate the independent effect of MVPA. Third, heterogeneity in frailty assessment tools (e.g., Fried phenotype versus FI scales) may also contribute to inconsistent findings, as some tools may be more sensitive to SB or LPA. Although pre-frailty was initially considered within our review scope, most included studies did not report pre-frailty-specific analyses separately from frailty or did not examine pre-frailty as an independent outcome. Therefore, we were unable to synthesize stage-specific evidence for pre-frailty. This limits the applicability of our findings to the early, potentially reversible stage of frailty progression.

In addition, several sources of bias inherent to evidence synthesis should be acknowledged. Publication and dissemination bias may be present because studies with statistically significant findings are more likely to be published and indexed, which may lead to an overrepresentation of stronger associations in the available literature. Selective outcome reporting in primary studies may also occur, as authors may preferentially report certain movement behavior metrics, model specifications, or frailty definitions based on statistical significance, which can result in an incomplete and potentially biased summary of the evidence. Moreover, although our searches were not restricted by language, it is still possible that some relevant non-English studies were missed during indexing, screening, or retrieval, which could introduce language-related bias. These issues should be considered when interpreting the overall strength and consistency of the findings.

## 6 Conclusion

This scoping review systematically synthesizes existing evidence on the relationship between 24-h movement behaviors and frailty. The findings underscore the importance of considering sleep, SB, LPA, and MVPA as an integrated whole across the 24-h day. MVPA emerges as the main protective factor for preventing and managing frailty, whereas prolonged ST, particularly continuous bouts exceeding 30 min, substantially increases frailty risk. Importantly, for older adults unable to meet MVPA recommendations, frequent and higher-intensity BST can still provide meaningful health benefits, offering a feasible and pragmatic intervention approach. However, substantial heterogeneity remains in measurement methods and frailty assessment tools across current studies, limiting direct comparison of the evidence. Future studies should prioritize standardization in measurement and reporting, including harmonizing accelerometer reporting protocols and defining thresholds for “prolonged sedentary bouts.” Longitudinal and intervention studies should be conducted using compositional data analysis and “time-reallocation” models to generate quantitative evidence that is interpretable and applicable in both clinical and policy contexts. Finally, evidence specific to pre-frailty remains limited because many studies did not report pre-frailty separately. Future work should explicitly target pre-frailty and frailty transitions to determine how 24-h time reallocation may support early prevention.

## Data Availability

The original contributions presented in the study are included in the article/Supplementary material, further inquiries can be directed to the corresponding authors.
