# Supplementary material for: Relationship between 24-h movement behaviors and frailty—a scoping review
Source: Front Public Health. 2026 Mar 19;14:1780746. doi: 10.3389/fpubh.2026.1780746 (PMC13043431; doi:10.3389/fpubh.2026.1780746)
Supplement: Supplementary file 1 [file Supplementary_file_1.docx]

Supplementary Material

# Appendix 1 – Search Strategy

Pubmed

| **1** | **((Sleep[MeSH Terms]) OR (sleep duration[Title/Abstract])) OR (sleep quality[Title/Abstract])** | 131684 |
| --- | --- | --- |
| **2** | **((Sedentary Behavior[MeSH Terms]) OR (sitting time[Title/Abstract])) OR (screen time[Title/Abstract])** | 21368 |
| **3** | **(((((((Motor Activity[MeSH Terms]) OR (Exercise[MeSH Terms])) OR (light physical activity[Title/Abstract])) OR (24-hour movement[Title/Abstract])) OR (24-hour activity[Title/Abstract])) OR (movement behavio[Title/Abstract])) OR (strenuous physical activity[Title/Abstract])) OR (high-intensity physical activity[Title/Abstract])** | 376278 |
| **4** | 1 OR 2 OR 3 | 507707 |
| **5** | **(((((Frailty[MeSH Terms]) OR (Frail Elderly[MeSH Terms])) OR (frail[Title/Abstract])) OR (prefrail[Title/Abstract])) OR (pre-frail[Title/Abstract])) OR (frailty syndrome[Title/Abstract])** | 36211 |
| **6** | 4 OR 5 | 2380 |

Web of Science

| **1** | **TS=(sleep OR "sleep duration" OR "sleep quality"**  **OR sedentar* OR "sitting time" OR "screen time" OR "sedentary behavior" OR "sedentary behaviour"**  **OR "light physical activity" OR "light-intensity physical activity" OR LPA OR LIPA OR "low-intensity physical activity"**  **OR "vigorous physical activity" OR "vigorous-intensity physical activity" OR "high-intensity physical activity" OR VPA OR "strenuous physical activity")** | **717987** |
| --- | --- | --- |
| **2** | **TI=(frail* OR "frailty" OR "frailty index" OR "clinical frailty scale" OR "frailty phenotype")** | **32684** |
| **3** | 1 AND 2 | **877** |

Embase

| **1** | **'sleep'/exp OR sleep:ti,ab OR 'sleep duration':ti,ab OR 'sleep quality':ti,ab OR 'sedentary behavior'/exp OR 'sedentary lifestyle'/exp OR sedentar*:ti,ab OR 'sitting time':ti,ab OR 'screen time':ti,ab OR 'motor activity'/exp OR 'exercise'/exp OR 'light physical activity':ti,ab OR 'light-intensity physical activity':ti,ab OR '24-hour movement':ti,ab OR '24-hour activity':ti,ab OR 'movement behavio*':ti,ab OR 'vigorous physical activity':ti,ab OR 'vigorous-intensity physical activity':ti,ab OR 'high-intensity physical activity':ti,ab OR 'strenuous physical activity':ti,ab)** | **1759043** |
| --- | --- | --- |
| **2** | **frail*:ti OR prefrail*:ti OR 'pre-frail':ti OR 'frailty index':ti OR 'clinical frailty scale':ti OR 'frailty phenotype':ti OR 'frailty syndrome':ti OR 'frail elderly':ti** | **30352** |
| **3** | 1 AND 2 | **3537** |

CINAHL

| **1** | **(MH "Sleep") OR TI sleep OR AB sleep OR TI "sleep duration" OR AB "sleep duration" OR TI "sleep quality" OR AB "sleep quality" OR (MH "Sedentary Behavior") OR TI sedentar* OR AB sedentar* OR TI "sitting time" OR AB "sitting time" OR TI "screen time" OR AB "screen time" OR (MH "Motor Activity") OR (MH "Exercise") OR TI "light physical activ*" OR AB "light physical activ*" OR TI "light-intensity physical activ*" OR AB "light-intensity physical activ*" OR TI "24-hour movement" OR AB "24-hour movement" OR TI "24-hour activity" OR AB "24-hour activity" OR TI "movement behavio*" OR AB "movement behavio*" OR TI "vigorous physical activ*" OR AB "vigorous physical activ*" OR TI "vigorous-intensity physical activ*" OR AB "vigorous-intensity physical activ*" OR TI "high-intensity physical activ*" OR AB "high-intensity physical activ*" OR TI "strenuous physical activ*" OR AB "strenuous physical activ*"** | **178307** |
| --- | --- | --- |
| **2** | **(TI frail* OR TI prefrail* OR TI "pre-frail" OR TI "frailty index" OR TI "clinical frailty scale" OR TI "frailty phenotype" OR TI "frailty syndrome" OR TI "frail elderly")** | **12591** |
| **3** | 1 AND 2 | **596** |
