# Supplementary material for: Relationship between 24-h movement behaviors and frailty—a scoping review
Source: Front Public Health. 2026 Mar 19;14:1780746. doi: 10.3389/fpubh.2026.1780746 (PMC13043431; doi:10.3389/fpubh.2026.1780746)
Supplement: Supplementary file 2 [file Supplementary_file_2.docx]

Supplementary Material

| **Table 1. Quality Assessment of the Included Cohort Studies** | | | | | | | | | | | | | | | | | | | | | |
| --- | --- | --- | --- | --- | --- | --- | --- | --- | --- | --- | --- | --- | --- | --- | --- | --- | --- | --- | --- | --- | --- |
| **Author/Year** | | **Were the two groups similar and recruited from the same population?** | **Was the exposure measured in the same way to assign participants to groups?** | | **Was the exposure measured in a valid and reliable way?** | | **Were potential confounding factors identified?** | | **Were strategies to deal with confounding factors stated?** | **Were the participants free of the outcome at the start of the study?** | | **Was the outcome measured in a valid and reliable way?** | | **Was the follow-up time sufficient and reported?** | **Was follow-up complete? If not, was it described and explored?** | | **Was an appropriate strategy used to address incomplete follow-up?** | | **Was statistical analysis appropriate?** | **Quality rating** | |
| Ensrud et al. 2012 | | Yes | Yes | | Yes | | Yes | | Yes | Yes | | Yes | | Yes | Yes | | Yes | | Yes | High quality | |
| Yuki et al. 2019 | | Yes | Yes | | Yes | | Yes | | Yes | Yes | | Yes | | Yes | Unclear | | Yes | | Yes | High quality | |
| Mañas et al. 2020 | | Yes | Yes | | Yes | | Yes | | Yes | No | | Yes | | Yes | Yes | | Yes | | Yes | High quality | |
| Guida et al. 2021 | | Yes | Yes | | Yes | | Yes | | Yes | No | | Yes | | Yes | Yes | | Yes | | Yes | High quality | |
| Mañas et al. 2020 | | Yes | Yes | | Yes | | Yes | | Yes | No | | Yes | | Yes | Yes | | Yes | | Yes | High quality | |
| Lefferts et al. 2021 | | Yes | Yes | | Yes | | Yes | | Yes | Yes | | Yes | | Unclear | Unclear | | Yes | | Yes | High quality | |
| Yang et al. 2025 | | Yes | Yes | | Yes | | Yes | | Yes | Yes | | Yes | | Yes | Unclear | | Yes | | Yes | High quality | |
| Lee et al. 2025 | | Yes | Yes | | Yes | | Yes | | Yes | Yes | | Yes | | Yes | Unclear | | Yes | | Yes | High quality | |
| **Table 2. Quality Assessment of the Included Cross-Sectional Studies** | | | | | | | | | | | | | | | | | | | | | |
| **Author/Year** | **Were inclusion criteria clearly defined?** | | | **Were the study subjects and setting described in detail?** | | **Was the exposure (activity behavior) measured in a valid and reliable way?** | | **Was the outcome (frailty) measured using objective criteria?** | | | **Were confounding factors identified?** | | **Were strategies to deal with confounding factors stated?** | | | **Was the outcome measured in a valid and reliable way?** | | **Was statistical analysis appropriate?** | | | **Quality rating** |
| Blodgett et al. 2014 | Yes | | | Yes | | Yes | | Yes | | | Yes | | Yes | | | Yes | | Yes | | | High |
| Mañas et al. 2017 | Yes | | | Yes | | Yes | | Yes | | | Yes | | Yes | | | Yes | | Yes | | | High |
| del Pozo-Cruz et al. 2017 | Yes | | | Yes | | Yes | | Yes | | | Yes | | Yes | | | Yes | | Yes | | | High |
| Huisingh-Scheetz et al. 2018 | Yes | | | Yes | | Yes | | Yes | | | Yes | | Yes | | | Yes | | Yes | | | High |
| Nagai et al. 2018 | Yes | | | Yes | | Yes | | Yes | | | Yes | | Yes | | | Yes | | Yes | | | High |
| Mañas et al. 2019 | Yes | | | Yes | | Yes | | Yes | | | Yes | | Yes | | | Yes | | Yes | | | High |
| Dantas da Silva et al. 2019 | Yes | | | Yes | | Yes | | Yes | | | Yes | | Yes | | | Yes | | Yes | | | High |
| Kehler et al. 2020 | Yes | | | Yes | | Yes | | Yes | | | Yes | | Yes | | | Yes | | Yes | | | High |
| Watanabe et al. 2020 | Yes | | | Yes | | Yes | | Yes | | | Yes | | Yes | | | Yes | | Yes | | | High |
| Higueras-Fresnillo et al. 2020 | Yes | | | Yes | | Yes | | Yes | | | Yes | | Yes | | | Yes | | Yes | | | High |
| Chen et al. 2020 | Yes | | | Yes | | Yes | | Yes | | | Yes | | Yes | | | Yes | | Yes | | | High |
| Lefferts et al. 2021 | Yes | | | Yes | | Yes | | Yes | | | Yes | | Yes | | | Yes | | Yes | | | High |
| Kikuchi et al. 2021 | Yes | | | Yes | | Yes | | Yes | | | Yes | | Yes | | | Yes | | Yes | | | High |
| Takamura et al. 2021 | Yes | | | Yes | | Yes | | Yes | | | Yes | | Yes | | | Yes | | Yes | | | High |
| Wanigatunga et al. 2022 | Unclear | | | Yes | | Yes | | Yes | | | Yes | | Yes | | | Yes | | Unclear | | | High |
| Li et al. 2022 | Yes | | | Yes | | Yes | | Yes | | | Yes | | Yes | | | Yes | | Yes | | | High |
| Martins et al. 2023 | Yes | | | Yes | | No | | Yes | | | Yes | | Yes | | | Yes | | Yes | | | High |
| Chang et al. 2023 | Yes | | | Yes | | No | | Yes | | | Yes | | Yes | | | Yes | | Yes | | | High |
| Yokote et al. 2023 | Yes | | | Yes | | Yes | | Yes | | | Yes | | Yes | | | Yes | | Yes | | | High |
| Li et al. 2024 | Yes | | | Yes | | Yes | | Yes | | | Yes | | Yes | | | Yes | | Yes | | | High |
| Tajima et al. 2024 | Yes | | | Yes | | Yes | | Yes | | | Yes | | Yes | | | Yes | | Yes | | | High |
| Yokote et al. 2024 | Yes | | | Yes | | Yes | | Yes | | | Yes | | Yes | | | Yes | | Yes | | | High |
| Meister et al. 2024 | Yes | | | Yes | | Yes | | Yes | | | Yes | | Yes | | | Yes | | Yes | | | High |
| Liu et al. 2025 | Yes | | | Yes | | Yes | | Yes | | | Yes | | Yes | | | Yes | | Yes | | | High |
| Nam et al. 2025 | Yes | | | Yes | | Yes | | Yes | | | Yes | | Yes | | | Yes | | Yes | | | High |
| Wingood et al.  2025 | Yes | | | Yes | | Yes | | Yes | | | Yes | | Yes | | | Yes | | Yes | | | High |
